# Evolutionary divergence of uric acid catabolic enzymes: structure–function constraints and genomic innovations across taxa

**DOI:** 10.1007/s12551-026-01466-3

**Published:** 2026-10-02

**Authors:** Júlia T. Rodrigues, Ana Carolina S. Batista, João Paulo M. S. Lima, Mariana T. Q. De Magalhães

**Affiliations:** 1 Programa de Pós-Graduação em Bioquímica e Imunologia, Instituto de Ciências Biológicas, Universidade Federal de Minas Gerais (UFMG), Belo Horizonte, MG 31270-901 Brazil; 2 Laboratório de Biofísica de Macromoléculas (LBM), Engenharia Química, Instituto Militar de Engenharia (IME), Rio de Janeiro, RJ 22290-270 Brazil; 3 BioinfoMetrópole (BioMe), Instituto Metrópole Digital (IMD), Universidade Federal Do Rio Grande Do Norte (UFRN), Natal, RN 59078-900 Brazil; 4 Programa de Pós-Graduação em Química, Instituto Militar de Engenharia (IME), Rio de Janeiro, Rio de Janeiro 22290-270 Brazil

**Keywords:** Uric acid catabolism, Gene fusion, Enzyme evolution, Transthyretin, Pseudoenzyme, Protein engineering

## Abstract

Uric acid degradation is a model for understanding how gene duplication, loss, and functional divergence drive metabolic evolution. In hominoids and uricotelic animals, uric acid is the terminal purine catabolite, whereas most other taxa further degrade it to allantoin and beyond. Comparative genomics and structural analyses reveal recurrent duplication and repurposing of key enzymes. 5-Hydroxyisourate hydrolase (HIUase) and transthyretin (TTR) share a common ancestral hydrolase, later diverging through neofunctionalization, while independent gene fusions reshaped the pathway, notably the gene fusion of *Urah* (HIUase) and *Urad* (2-oxo-4-hydroxy-4-carboxy-5-ureidoimidazoline decarboxylase, or OHCUd) into single-chain allantoin synthases in plants, diatoms, and bacteria. Elastic network modeling this gene fusion shows uncorrelated motions between the catalytic domains, suggesting its advantage stems from fixed stoichiometry and spatial proximity rather than conformational coupling. Further divergence is seen in enzyme families with complementary substrate specificities, and gene clustering in bacteria and yeast reflects regulatory optimization under nitrogen limitation, paralleling the ureide pathway in legumes. In hominoids, pseudogenization of urate oxidase relaxed constraints on downstream enzymes, elevating serum uric acid, an ancestral adaptation now linked to hyperuricemia. Gut microbiome-mediated degradation and links between Urad variants and cancer risk underscore the pathway’s continuing relevance. Mammalian Urad orthologs show strong sequence conservation. Molecular modeling of the human ortholog demonstrated a conserved fold, consistent with a predominantly α-helical secondary structure content supported by circular dichroism, whereas kinetic characterization revealed the absence of detectable catalytic activity. Building on these principles, evolution-guided protein engineering achieved partial functional interconversion between transthyretins and HIUases without disrupting the ancestral scaffold, revealing purine catabolism’s plasticity as a model for enzyme innovation and the metabolism-disease-protein design interface.

## Biophysical and computational approaches used in this study

This review uses an integrative framework that combines biophysical analysis, comparative genomics, structural biology, and evolutionary approaches to investigate the diversification of uric acid catabolic enzymes. Experimentally determined protein structures from the Protein Data Bank were analyzed through structural superposition to assess fold conservation, oligomeric organization, and domain architecture, with structural similarity measured by root mean square deviation (RMSD). Detailed comparisons of active-site architectures, including bimetallic catalytic centers, revealed residue-level determinants of substrate specificity, as illustrated by allantoate amidohydrolase and ureidoglycolate amidohydrolase. Natural fusion proteins, such as HIUase and OHCUd, provide additional insight into domain assembly, interdomain compatibility, and the spatial relationships between catalytic sites. Sequence alignments and phylogenetic analyses identified conserved motifs, quantified sequence similarity, reconstructed evolutionary histories, and examined convergent events, including independent gene fusions. Comparative genomic analyses further examined intron–exon conservation, gene duplication patterns, genomic context, and gene clustering, offering insights into pathway organization and potential regulatory coupling. Finally, genome mining and SNP association studies were used to assess the persistence and potential biomedical relevance of uric acid catabolic genes in the human genome.

Sequence conservation across mammalian URADd orthologs was assessed through Shannon entropy analysis calculated from a multiple sequence alignment of representative mammalian sequences retrieved from UniProtKB. For each alignment position, entropy was computed as *H* = −∑ *p* log_2_*p*, where *p* denotes the frequency of each amino acid state, with a theoretical maximum of log_2_(21) bits used as reference. To examine structural conservation independently of sequence divergence, pairwise RMSD values were calculated from structural superpositions of experimentally determined or predicted URADd structures across mammals. The resulting distance matrix was subjected to principal coordinate analysis (PCA) to visualize the structural landscape occupied by mammalian orthologs, and to hierarchical clustering using average linkage to assess correspondence between structural and phylogenetic relationships. Active-site cavity analysis was also performed on structural models of OHCUd orthologs and related proteins to evaluate whether changes in cavity geometry accompany the functional attenuation observed in human OHCUd. Cavity detection was performed using the Find Cavities tool in ChimeraX (pyKVFinder).

Together, these complementary approaches establish a coherent framework linking molecular architecture, evolutionary innovation, and physiological outcomes.

### Nomenclature note

Throughout this review, two parallel naming conventions are used for the same set of catalytic activities in the uricolytic pathway, reflecting different traditions in the literature. *Uox*, *Urah*, and *Urad* are gene symbols originally established in animal and yeast genomics (e.g., zebrafish, *Drosophila*, and primate genome annotations) for urate oxidase, 5-hydroxyisourate hydrolase (HIUase), and 2-oxo-4-hydroxy-4-carboxy-5-ureidoimidazoline (OHCUd) decarboxylase, respectively. The same three activities are more broadly known by their enzymatic names: uricase (urate oxidase, EC 1.7.3.3), HIUase (5-hydroxyisourate hydrolase, EC 3.5.2.17), and OHCU decarboxylase (EC 4.1.1.97). In this review, we favor the gene symbol when discussing genomic or phylogenetic context (Genomic organization of uric acid degradation genes and Ecological, evolutionary, and biomedical contexts of uric acid metabolism sections) and the functional name when discussing biochemistry and structure (Purine catabolism and uric acid degradation: an overview and Functional divergence and evolutionary innovation in uric acid degradation enzymes sections). Bacterial and plant orthologs follow locus-specific nomenclature (e.g., PucL/PucM in *Bacillus subtilis*, UGLYAH in *Arabidopsis thaliana*) and are introduced individually where first discussed (Functional divergence between UGLYAH and UGLYAH2 proteins section, Genomic organization of uric acid degradation genes section).

## Purine catabolism and uric acid degradation: an overview

**Fig. 1:**
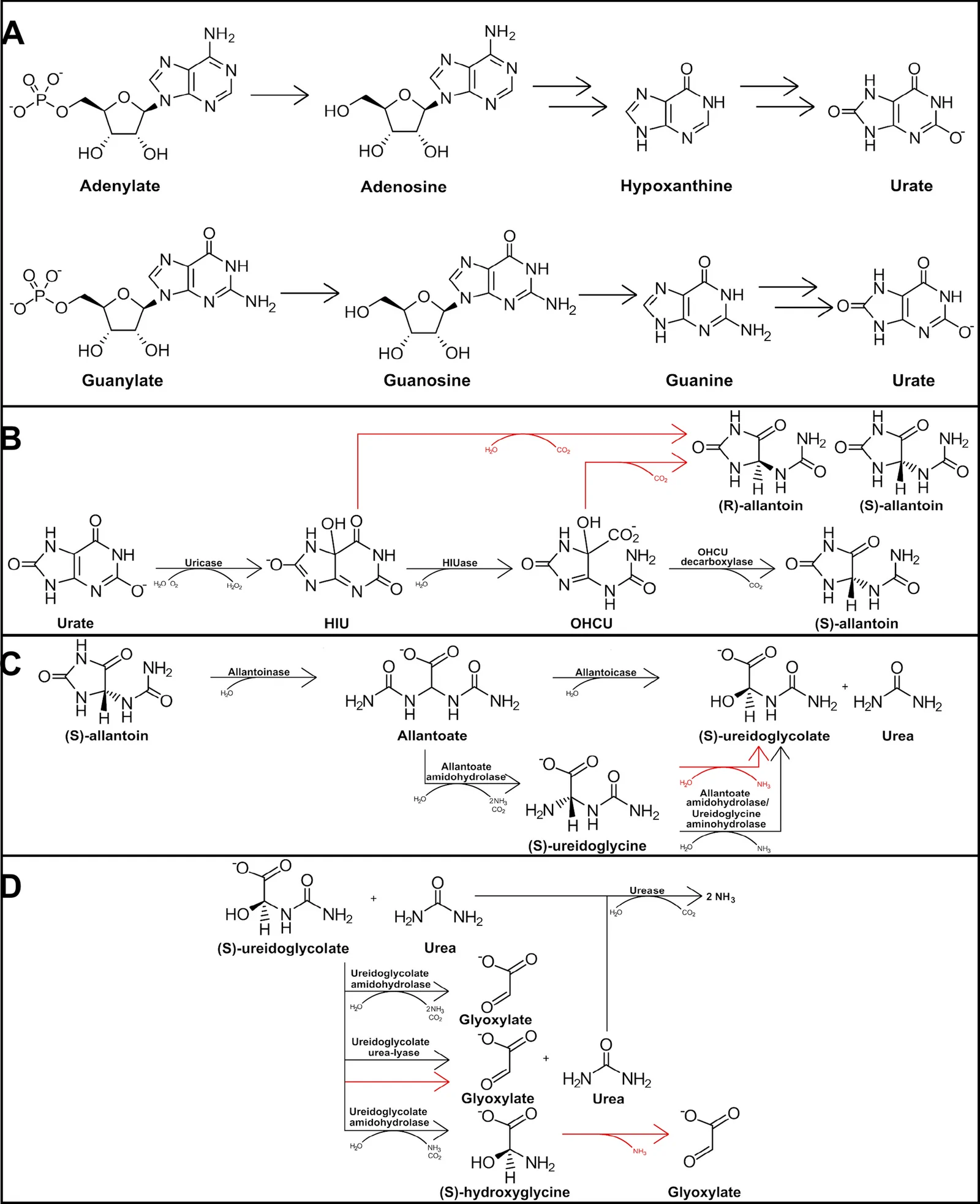
Overview of purine catabolism and uric acid degradation. **A** Formation of uric acid from adenylate- and guanylate-derived metabolites; red arrows indicate spontaneous, non-enzymatic decomposition reactions. **B** Sequential conversion of uric acid to (S)-allantoin by urate oxidase, 5-hydroxyisourate hydrolase (HIUase), and OHCUd decarboxylase (OHCUd). **C** Alternative routes of allantoate degradation to ureidoglycolate via allantoicase or allantoate amidohydrolase. **D** Alternative routes of ureidoglycolate degradation to glyoxylate via ureidoglycolate amidohydrolase or ureidoglycolate urea-lyase; when urease is present, urea is further hydrolyzed to ammonium and carbon dioxide

During purine nucleotide catabolism, adenosine monophosphate (AMP) and guanosine monophosphate (GMP) are progressively degraded through dephosphorylation, cleavage of the ribose moiety, and enzymatic modification of the resulting purine bases. Although different routes may contribute to these transformations, the pathway ultimately converges on the formation of uric acid, as summarized in Fig. 1A (Salway 2018).

In hominoids and many uricotelic^1^ animals, including birds, reptiles, and terrestrial insects, uric acid is the final product of purine catabolism (Keebaugh and Thomas 2010; Salway 2018). Because uric acid can be eliminated with minimal water loss, its excretion is an efficient strategy for nitrogen disposal in animals adapted to water-limited environments. In hominoids specifically, this outcome reflects the evolutionary loss of functional urate oxidase (*UOX*; uricase) rather than an ancestral uricotelic strategy. The inactivation of *uricase* interrupted the enzymatic conversion of uric acid to allantoin and may have altered the selective constraints on downstream components of the uricolytic pathway.

By contrast, in most placental mammals, as well as amphibians, fish, marine invertebrates, plants, fungi, and many microorganisms, uric acid is further metabolized to (S)-allantoin through the sequential activity of urate oxidase, HIUase, and OHCUd (Ramazzina et al. 2006). Urate oxidase catalyzes the oxidation of urate to 5-hydroxyisourate (HIU), which is subsequently hydrolyzed by HIUase to generate OHCU. OHCUd then catalyzes the stereoselective decarboxylation of OHCU, yielding (S)-allantoin (Fig. 1B). These enzymes accelerate the conversion of chemically labile intermediates and ensure the stereoselective formation of the biologically relevant enantiomer (Holmberg 1939; Przylecki 1928; Kaltwasser 1968; Scazzocchio and Darlington 1968, Bongaerts 1978).

The OHCUd-catalyzed decarboxylation is mechanistically unusual: unlike most decarboxylases, which rely on cofactors such as pyridoxal-5′-phosphate or thiamine pyrophosphate to accommodate the electrons released upon carbon–carbon bond cleavage, OHCUd operates without any cofactor, a rare strategy. Structural and kinetic studies of the *Klebsiella pneumoniae* enzyme indicate that substrate protonation at N6 provides a driving force for CO_2_ release, while a conserved glutamine residue inductively destabilizes the C5–carboxylate bond through hydrogen bonding, analogous to the electron-withdrawing mechanism of purine nucleoside phosphorylase rather than the ground-state electrostatic destabilization used by OMPDC (French and Ealick, 2011). The resulting enolate intermediate is stabilized by resonance within the hydantoin ring itself, which thus serves as an intramolecular electron sink. A conserved active-site histidine subsequently mediates stereospecific proton transfer, tautomerizing this intermediate exclusively to (S)-allantoin (Cendron et al., 2007; French and Ealick 2010a, b).

Beyond the three-enzyme uricolytic core, in amphibians, fish, marine invertebrates, plants, and microorganisms, allantoin does not necessarily represent the end point of the pathway. It can be converted to allantoate by allantoinase and subsequently degraded to ureidoglycolate through distinct enzymatic routes (Fig. 1C). In animals, fungi, and many bacteria, allantoicase converts allantoate into ureidoglycolate and urea. In plants and some bacteria, allantoate amidohydrolase generates ureidoglycine, carbon dioxide, and ammonium. Ureidoglycine is an unstable intermediate that may undergo spontaneous decomposition; however, in several organisms, a dedicated ureidoglycine aminohydrolase catalyzes its conversion to ureidoglycolate and ammonium (Agarwal et al. 2007; Shin et al. 2014).

Ureidoglycolate can also be metabolized through alternative routes (Fig. 1D). Ureidoglycolate amidohydrolase catalyzes its conversion to glyoxylate, ammonium, and carbon dioxide, potentially through the formation of an unstable hydroxyglycine intermediate. Alternatively, ureidoglycolate urea-lyase generates glyoxylate and urea. When urease is present, the resulting urea can be further hydrolyzed to ammonium and carbon dioxide (Percudani et al. 2013; Shin et al. 2014; Werner et al. 2010).

The chemical instability of the uricolytic intermediates has been recognized since early biochemical investigations of urate oxidase. Canellakis and Cohen (1955) provided evidence for the formation of at least three unstable intermediates during uric acid oxidation by uricase, including a compound designated UIDC (5-ureido-2-imidazolidone-4,5-diol-4-carboxylic acid), which corresponds to the OHCU stage of the pathway. UIDC was shown to decompose asymmetrically and nonenzymatically to urea and alloxanic acid and could be recovered intact only when stabilized by borate buffer through the formation of a borate complex with its adjacent hydroxyl groups; in the presence of phosphate buffer, only its degradation products were recovered. Spectrophotometric evidence for a preceding unstable intermediate with maximal absorption between 300 and 330 nm (corresponding to HIU) had been reported even earlier (Praetorius, 1948, as cited in Canellakis and Cohen 1955).

The metastable nature of HIU was subsequently characterized in mechanistic detail by Altarsha et al. (2009), who described 5-hydroxyisourate as an unstable species produced during the first step of urate oxidation and subject to spontaneous decomposition to allantoin in the absence of enzymatic activity. Together, these observations establish that both HIU and OHCU are chemically labile species whose efficient processing requires dedicated enzymatic machinery, a constraint that shaped the evolution of the complete uricolytic pathway and remains directly relevant to the design of multienzyme therapeutic systems. This is not merely a biochemical abstraction: mice deficient in HIU hydrolase develop hepatomegaly and hepatocellular carcinoma, providing direct in vivo evidence that unprocessed uricolytic intermediates carry genuine toxicological consequences, with direct relevance to the clinical use of exogenous urate oxidase (Stevenson et al. 2010).

Together, these reactions illustrate the progressive diversification of purine catabolism across different organisms. The broad conservation of cofactor-independent UOX across plants, fungi, and animals is consistent with an ancient origin of the oxidative branch within aerobic eukaryotes (Marchetti et al. 2016); however, because this branch requires molecular oxygen as substrate, it cannot predate the Great Oxidation Event (Lyons et al. 2014), and oxygen-independent reductive routes described in anaerobic bacteria (Iwadate and Kato 2019; Li et al. 2025) may represent evolutionarily older, though mechanistically distinct, strategies for purine nitrogen catabolism. Although the conversion of uric acid to (S)-allantoin is relatively conserved in species that retain the uricolytic pathway, the downstream metabolism of allantoin and ureidoglycolate proceeds through alternative enzymatic routes. This diversity is reflected not only in the presence or absence of specific enzymes, but also in gene loss, gene duplication, genomic clustering, and the emergence of natural fusion proteins. These evolutionary events provide a useful framework for investigating how molecular architecture and genomic organization have shaped the functional diversification of uric acid catabolism.

## Functional divergence and evolutionary innovation in uric acid degradation enzymes

The diversification of uric acid catabolism provides several examples of how metabolic pathways evolve through gene duplication, neofunctionalization, domain fusion, gene loss, and active-site remodeling. Although the enzymes involved in this pathway catalyze reactions within a common metabolic context, their evolutionary histories reveal distinct molecular strategies for functional adaptation. The following sections highlight representative cases in which structural, biochemical, phylogenetic, and genomic analyses provide complementary insights into the evolution of uric acid degradation.

### HIUase and transthyretin: structural and functional links

Although urate oxidase has been investigated for nearly a century, the enzymatic role HIUase was established much later, particularly through studies published around the turn of the twenty-first century. Early reports demonstrated HIUase activity in soybean (*Glycine max*) and examined the coding regions of HIUase-related genes in microorganisms such as *Bacillus subtilis* (Sarma et al. 1999; Schultz et al. 2001).

In the first structural and functional studies, HIUase-related proteins were designated transthyretin-related or transthyretin-like proteins (TRP/TLP) because of their remarkable structural resemblance to transthyretin (TTR), the vertebrate thyroid hormone transport protein (Lee et al. 2005; Hennebry et al. 2006a; Jung et al. 2006). Both protein families exhibit a predominantly *β*-sheet-rich architecture and commonly assemble as homotetramers (Richardson 2015; Carrijo de Oliveira et al. 2021). Despite their distinct biological roles, representative HIUases and TTRs also retain detectable sequence similarity, with pairwise comparisons generally revealing approximately 25–30% sequence identity (Hennebry et al. 2006b).

**Fig. 2:**
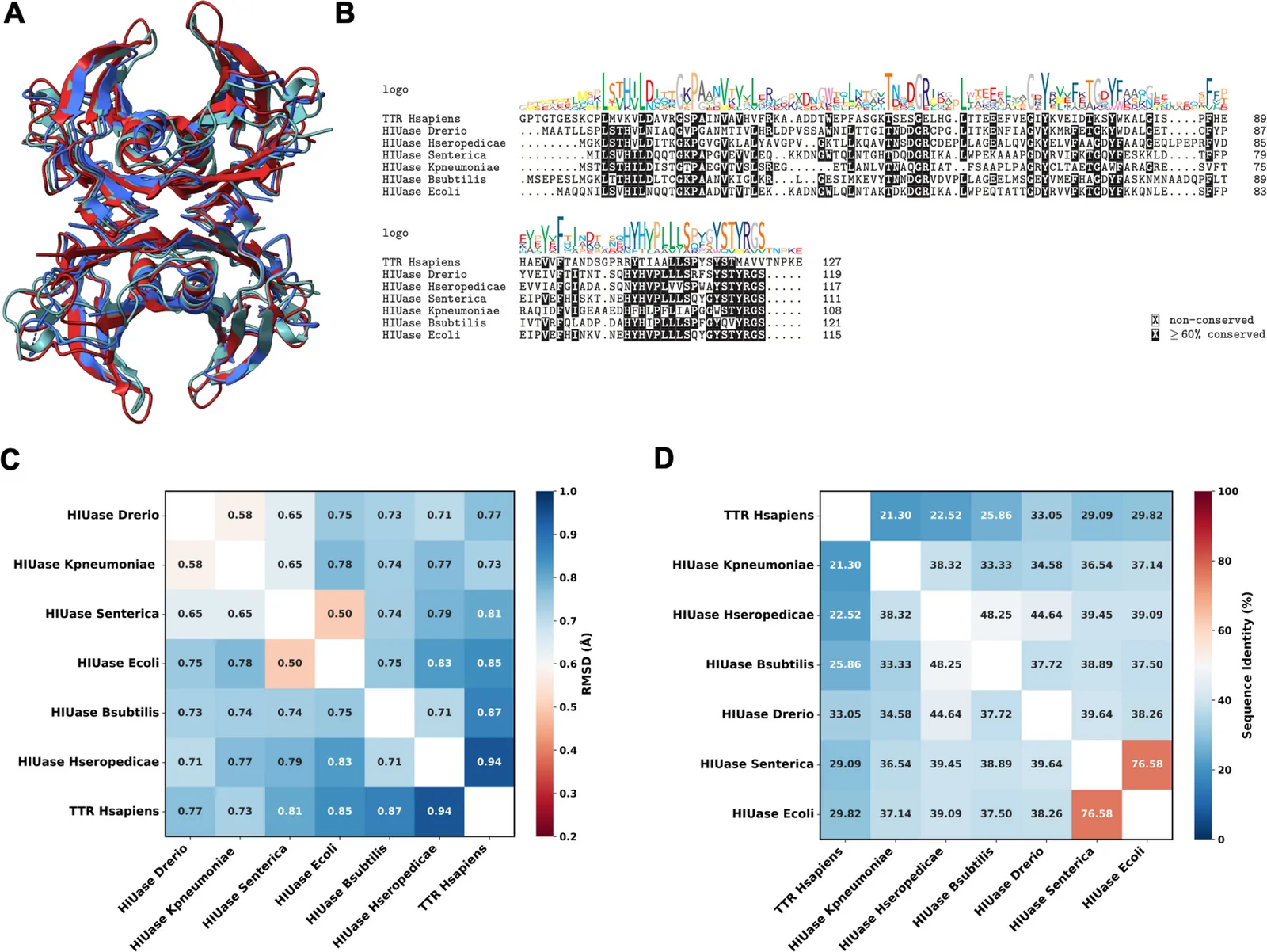
Structural relationship between transthyretin and HIUase. **A** Superposition of the tertiary structures of human TTR (PDB ID: 1FHN, red), *K. pneumoniae* HIUase (PDB ID: 3QVA, dark blue), and *H. seropedicae* HIUase (PDB ID: 8UW4, light blue). **B** Sequence alignment between human TTR and representative HIUases. **C** Matrix of RMSD values from structural comparisons of human TTR (PDB ID: 1FHN) with HIUases from *K. pneumoniae* (3QVA), *Danio rerio* (2H1X), *Escherichia coli* (2IGL), *Bacillus subtilis* (2H0E), *Salmonella enterica* (2GPZ), and *H. seropedicae* (8UW4). **D** Matrix of sequence identity values from pairwise comparisons between human TTR and the selected HIUases

This relationship becomes particularly evident when the primary and tertiary structures of human TTR are compared with those of representative HIUases deposited in the Protein Data Bank (PDB) (Sebastião et al. 2001; French and Ealick 2011; Hennebry et al. 2006a; Jung et al. 2006; Zanotti et al. 2006; Lemos et al., 2024). Several residues are conserved across the two protein families (Fig. 2B and D). Structural superpositions performed for the proteins included in Fig. 2 reveal low root mean square deviation (RMSD) values, ranging from 0.731 Å for human TTR and *Klebsiella pneumoniae* HIUase to 0.942 Å for human TTR and *Herbaspirillum seropedicae* HIUase. These results highlight the close structural relationship between TTR and HIUase despite their functional divergence (Fig. 2A and C).

This structural conservation extends beyond overall fold similarity: HIUase and TTR share the same prealbumin-like β-sandwich architecture, assembling into tetramers around a central hydrophobic channel. In HIUase, this channel constitutes the active site, lined by ten residues (His12, Asp50, Arg52, His103, Pro105, Leu107, Tyr116, Arg117, Gly118, and Ser119) that are highly conserved across bacterial and eukaryotic HIUases. Nine of these ten residues are replaced by nonconservative substitutions in TTR, yet only two (Y116T and I16A) appear sufficient to drive the enzyme-to-transporter transition. Tyr116, the first residue of the HIUase signature tetrapeptide YRGS, sterically occludes the channel; its substitution alone abolishes ~99% of catalytic activity. The additional I16A substitution releases the neighboring Leu107 side chain, opening a cavity large enough to accommodate thyroxine. The resulting double mutant is both catalytically dead and capable of binding thyroxine with an affinity profile closely resembling native human TTR (Cendron et al. 2011). The identification of TTR genes in lamprey indicates that the underlying gene duplication predates the divergence of Agnatha from Gnathostomata, over 530 million years ago, placing the origin of TTR at the base of vertebrate evolution, prior to the diversification of all extant vertebrate lineages (Cendron et al. 2011).

Comparative analyses of vertebrate TTR- and HIUase-related genes further revealed conservation of their intron–exon organization, supporting the hypothesis that these proteins share a common ancestor. The most widely accepted evolutionary model proposes that an ancestral HIUase gene underwent duplication early in chordate evolution (Prapunpoj et al. 2000; Zanotti et al. 2006; Carrijo de Oliveira et al. 2021; Yamauchi and Kasai 2018). Following duplication, the accumulation of mutations in one gene copy altered residues associated with catalysis while preserving the β-rich structural scaffold. These changes were associated with the emergence of thyroid hormone-binding sites and the functional specialization of TTR as a transport protein (Fig. 2).

Additional diversification events have also occurred within the HIUase-related family. In *Arabidopsis thaliana*, alternative splicing gives rise to three transthyretin-like protein (TTL) variants, including two cytoplasmic isoforms and one peroxisomal isoform (Pessoa et al. 2010). The full-length *A. thaliana* TTL protein contains an N-terminal domain related to OHCU decarboxylase and a C-terminal HIUase-related domain. Biochemical characterization demonstrated that this naturally fused protein is bifunctional in vitro, displaying both HIUase and OHCU decarboxylase activities (Pessoa et al. 2010). Subsequent studies identified similar Urah–Urad fusion architectures in additional organisms, including plants and diatoms (Oh et al. 2018).

The structural and functional relationship between HIUase and TTR illustrates how gene duplication followed by limited sequence changes can preserve a stable protein scaffold while enabling a profound functional transition from enzymatic catalysis to ligand transport. Studies exploring the partial functional interconversion of HIUase- and TTR-related proteins further reinforce the value of evolutionary analyses for guiding structural and biochemical investigations (Cendron et al. 2011; Lemos et al., 2024).

### Urah–Urad gene fusions as metabolic and evolutionary innovations

Gene fusion is an important mechanism of evolutionary innovation. It can generate multifunctional proteins by combining domains that are encoded separately in other organisms. In metabolic pathways, such events may promote coordinated expression, fixed domain stoichiometry, and spatial colocalization of consecutive catalytic activities. The occurrence of a fusion protein can therefore provide evidence of a functional relationship between the corresponding domains.

The conversion of 5-hydroxyisourate (HIU) to (*S*)-allantoin provides a clear example of this principle. In many organisms, HIU hydrolase and OHCUd decarboxylase act as independent enzymes in two consecutive reactions. However, they also occur as a single polypeptide in several plants, diatoms, and some bacteria. These naturally fused proteins are sometimes referred to as allantoin synthases. In *A. thaliana*, for example, the N-terminal region corresponds to the OHUCd domain, while the C-terminal region corresponds to HIUase, thereby combining the two catalytic activities within a single protein (Pessoa et al. 2010; Oh et al. 2018).

Phylogenetic analyses indicate that HIUase-OHCUd fusions arose independently in different lineages, providing an example of convergent evolution (Oh et al. 2018). The repeated emergence of these proteins is consistent with the chemical instability of HIU and OHCUd established by early biochemical studies (Canellakis and Cohen 1955; Altarsha, Castro, and Monard, 2009; see Purine catabolism and uric acid degradation: an overview section), which undergo spontaneous decomposition in the absence of enzymatic catalysis. By ensuring fixed stoichiometry, coordinated expression, and spatial proximity between the two catalytic domains, fusion may facilitate efficient processing of unstable intermediates.

Structural analyses of natural fusion proteins provide additional insight into this evolutionary strategy. Available structural data indicate that the Urah domains form a tetrameric core, while the HIUase domains are organized as dimers associated with the surface of this assembly (Oh et al. 2018). The catalytic sites are separated by approximately 40 Å, and direct substrate channeling has not been demonstrated. This structural observation, however, is derived from an isolated, crystallized protein and does not exclude the possibility of higher-order assembly under physiological conditions. We hypothesize that the uricolytic pathway particularly in organisms where Urah and Urad occur as natural fusions alongside urate oxidase may be organized, in vivo, into a larger transient multienzyme assembly analogous to the purinosome described for de novo purine biosynthesis (An, Kyoung, and Benkovic, 2010), whose component enzymes likewise include natural multi-domain fusions such as PAICS (He J et al, 2022). This possibility is, at present, speculative and unsupported by direct experimental evidence in the uricolytic system; however, given the chemical instability of the HIU and OHCU intermediates discussed throughout this review, the potential fitness benefit of minimizing their diffusional loss through such an assembly would be considerable. We therefore propose that in vivo approaches capable of detecting transient or condition-dependent protein–protein interactions such as proximity-labeling techniques, live-cell fluorescence-based interaction assays, or co-immunoprecipitation under varying nitrogen or purine availability represent an important and currently unexplored direction for testing this hypothesis. Instead, the fusion architecture appears to promote colocalization of consecutive enzymatic activities and may reduce intermediate loss without requiring direct channeling. The limited interdomain interface also suggests that steric compatibility may have been more important than extensive direct contacts during the independent emergence of these fusion proteins.

The physiological context of *Urah–Urad* fusion is particularly relevant in plants and nitrogen-fixing bacteria. In legumes, ureide metabolism contributes to the assimilation and long-distance transport of fixed nitrogen. Uric acid-derived metabolites have also been associated with plant responses to environmental stresses, including drought, salinity, and high light. In bacteria involved in symbiotic nitrogen fixation, the metabolism of purine-derived nitrogen compounds may also be advantageous within nutrient-limited environments. However, although these observations provide a plausible physiological context for the emergence of *Urah–Urad* gene fusions, the specific adaptive benefit of fusion under each condition remains to be demonstrated experimentally.

The recurrent appearance of *Urah–Urad* fusions across phylogenetically distant organisms indicates that the integration of consecutive catalytic activities has repeatedly been favored during the evolution of the uricolytic pathway. These naturally occurring proteins may also inspire the design of engineered multifunctional enzymes. These architectures provide both evolutionary validation and structural blueprints for the design of single-chain multienzyme therapeutics targeting the complete uricolytic pathway.

### Functional divergence between UGLYAH and UGLYAH2 proteins

Not all cases of functional divergence in the uricolytic pathway involve structural remodeling or domain fusion. The UGLYAH protein family illustrates how changes in genomic context and pathway organization alone can drive the emergence of distinct enzymatic functions from a common ancestral scaffold.

A clear example of functional divergence has been described within the ureidoglycine aminohydrolase (UGLYAH) protein family. Two functional subgroups have been identified. UGLYAH proteins release ammonium from (S)-ureidoglycine, the unstable product of allantoate amidohydrolase (AAH), but do not degrade allantoate. In contrast, UGLYAH2 proteins act on both (S)-ureidoglycine and allantoate, releasing ammonium from the former and urea from the latter (French and Ealick 2010a, b).

Comparative genomic analyses revealed distinct associations between these enzymes and other pathway components. In the genomes analyzed, UGLYAH genes consistently co-occurred with AAH genes, whereas UGLYAH2 genes were mutually exclusive with AAH. Because ureidoglycine is generated by AAH, this distribution led to the hypothesis that UGLYAH2 proteins primarily function as allantoicases, with ureidoglycine hydrolysis as an accessory activity (Puggioni et al. 2014).

Two evolutionary scenarios have been proposed to explain the divergence between UGLYAH and UGLYAH2. In the gene-gain/function-loss model, allantoate degradation with urea release represents the ancestral activity. Ureidoglycine hydrolysis initially existed as a secondary activity with limited physiological relevance. The emergence of AAH created a metabolic context in which ureidoglycine hydrolysis became advantageous by promoting urease-independent nitrogen recycling. Subsequent loss of allantoicase activity gave rise to specialized UGLYAH proteins. Alternatively, in the function-gain/gene-loss model, ureidoglycine hydrolysis represents the ancestral activity, whereas allantoate degradation emerged later. In this scenario, the acquisition of allantoicase activity rendered AAH redundant, resulting in its loss from genomes carrying UGLYAH2 (Puggioni et al. 2014).

Neither model requires gene duplication. Both assume a transitional state in which alternative pathway branches coexisted (Magadum et al. 2013; Moore and Purugganan 2003). The genomic association between UGLYAH2 and urease further supports the functional interpretation of these enzymes, as urease is required for hydrolyzing the urea generated from allantoate. Conversely, organisms carrying UGLYAH often lack urease, consistent with direct ammonium release from ureidoglycine. Biochemical evidence favors the gene-gain/function-loss model, although the phylogenetic distribution of these proteins does not fully resolve the sequence of evolutionary events (Puggioni et al. 2014).

This example shows how enzyme function can diverge through changes in pathway organization and genomic context, even without gene duplication. It also demonstrates the importance of integrating biochemical characterization with comparative genomics when assigning physiological roles to homologous proteins.

### Allantoate amidohydrolase and ureidoglycolate amidohydrolase: structural determinants of substrate specificity

Another example of functional divergence within the uric acid degradation pathway involves allantoate amidohydrolase (AAH) and ureidoglycolate amidohydrolase (UAH). These homologous enzymes display high structural similarity and share approximately 30% sequence identity (Shin et al. 2014). Both catalyze the cleavage of an amide bond, and their substrates are chemically related, differing mainly because allantoate contains an additional ureido group. Despite these similarities, the enzymes exhibit strict substrate specificity: AAH acts on allantoate, while UAH hydrolyzes ureidoglycolate.

**Fig. 3:**
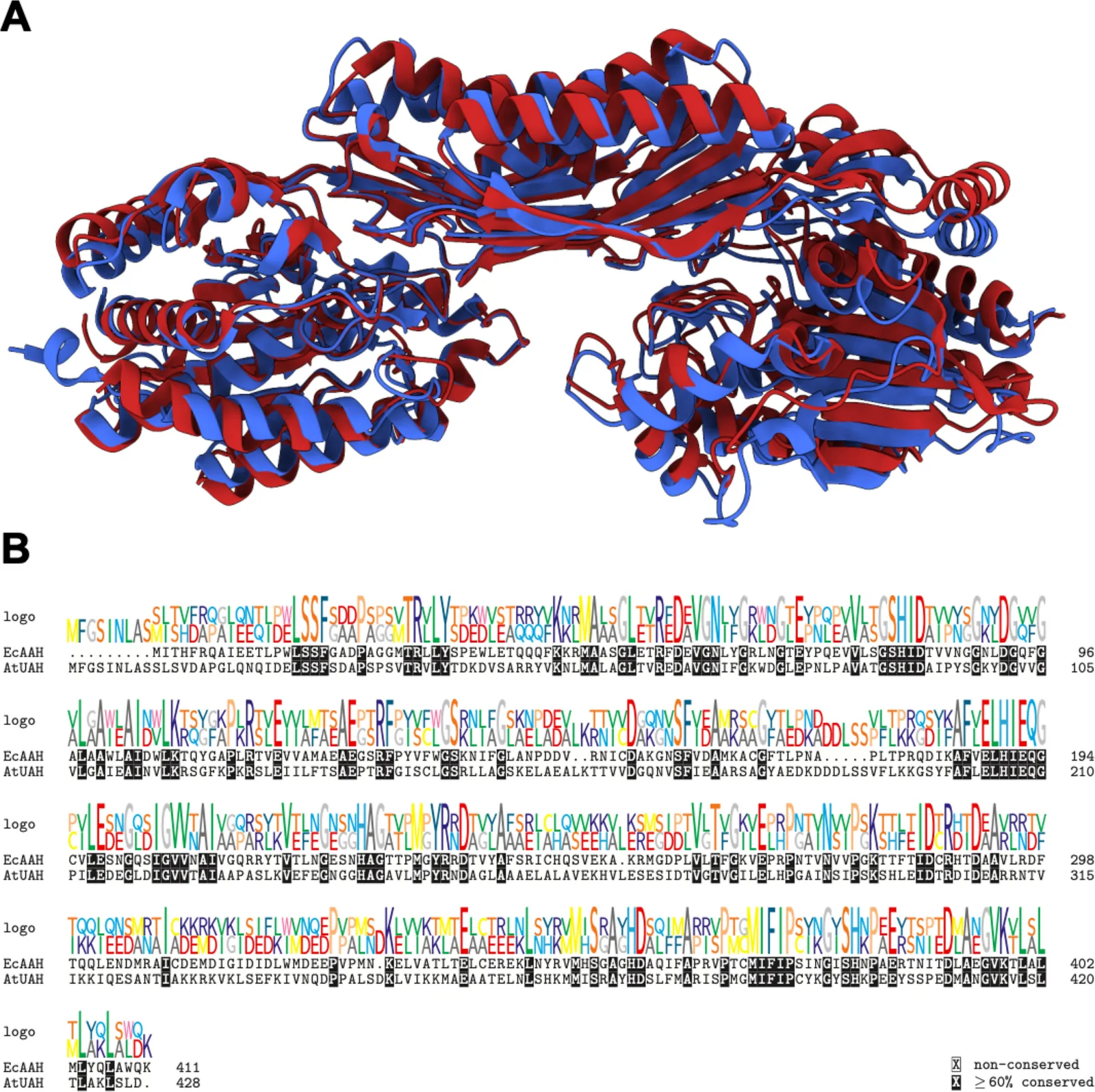
Structural comparison between allantoate amidohydrolase and ureidoglycolate amidohydrolase. **A** Superposition of the tertiary structures of *E. coli* allantoate amidohydrolase (AAH; PDB ID: 4PXD, red) and *A. thaliana* ureidoglycolate amidohydrolase (UAH; PDB ID: 4PXB, blue). **B** Sequence alignment between *E. coli* AAH and *A. thaliana* UAH, highlighting residues involved in substrate recognition and the QXR motif characteristic of AAH

Structural studies of *A. thaliana* UAH revealed how subtle differences within a conserved catalytic scaffold determine substrate recognition (Fig. 3A). UAH contains a bimetallic catalytic center and adopts distinct ligand-bound conformations during catalysis. Comparisons of substrate-, intermediate-, and product-bound structures indicate that substrate recognition depends not only on the presence of catalytic residues but also on the geometry and conformational dynamics of the active site (Shin et al. 2014).

The bimetallic centers found across this branch of the uricolytic pathway are not structurally uniform, despite superficial similarity in their catalytic strategy of metal-activated hydrolysis. Allantoinase, which acts upstream of AAH and UAH, retains the classical (β/α)_8_ TIM-barrel fold of the amidohydrolase superfamily, with its binuclear metal center bridged by a post-translationally carbamylated lysine residue (Kim K et al. 2009, PDB 3E74). Functional analyses in that same study showed that zinc occupancy at this metal center is essential not only for catalysis but also for the enzyme’s enantioselectivity toward its substrate, distinguishing canonical Zn-dependent allantoinase from a more recently identified metal-independent allantoinase variant. AAH, by contrast, adopts an α/β scaffold homologous to the M20 family of zinc exopeptidases and lacks the TIM-barrel architecture altogether; its two co-catalytic Zn^2+^ ions, together with the bound substrate, dictate specificity for N-carbamyl bond hydrolysis over peptide bond hydrolysis (Agarwal et al 2007). UAH shares the AAH fold rather than the allantoinase fold, consistent with the two enzymes’ close structural and mechanistic homology, but coordinates a binuclear Mn^2+^ center instead of Zn^2+^ (Shin I, Han K, Rhee S 2014, PDB 4PXB). Ligand binding to UAH induces closure of a hinge region over the catalytic domain, positioning each reaction intermediate distinctly within the bimetal active site. It is worth noting that metal identity in this enzyme family is not always unambiguous across crystallographic studies—structurally homologous active sites have been reported with either Mn^2+^ or Zn^2+^ occupying equivalent positions, likely reflecting metal promiscuity or purification/crystallization conditions rather than a strict, evolutionarily fixed metal preference. Where relevant, we report the metal identity as validated by the original structural studies cited.

A particularly relevant difference involves Tyr423 in UAH. This residue restricts ligand positioning and contributes to the productive orientation of ureidoglycolate through interactions with neighboring residues. In AAH, the equivalent position is occupied by glycine, which creates additional space for the pro-S ureido group of allantoate. This group is further stabilized by a QXR motif characteristic of AAH (Fig. 3B). Structural analyses and mutagenesis also indicate that both enzymes undergo an open-to-closed conformational transition upon ligand binding, highlighting the importance of active-site architecture and conformational flexibility in substrate discrimination (Shin et al. 2014).

The comparison between AAH and UAH shows how a small number of substitutions within a conserved structural framework can alter active-site volume, ligand interactions, and conformational behavior. These relatively subtle molecular changes are sufficient to confer strict specificity for chemically related substrates.

Together, these examples show that the evolution of uric acid catabolism reflects distinct, complementary mechanisms: scaffold repurposing (HIUase/TTR), convergent domain fusion (HIUase-OHCUd), branch reorganization without duplication (UGLYAH/UGLYAH2), and active-site remodeling through few substitutions (AAH/UAH).

## Genomic organization of uric acid degradation genes

The evolution of uric acid catabolism involves not only changes in protein sequence and enzyme activity but also modifications in the genomic organization of pathway components. In several microorganisms, genes encoding transporters, enzymes, and regulatory proteins involved in purine degradation are physically clustered or coordinately regulated as documented for the puc system of *Bacillus subtilis* (Schultz et al. 2001), the hpx cluster of *Klebsiella pneumoniae* (De la Riva et al. 2008; Guzmán et al. 2011), and the DAL cluster of *Saccharomyces cerevisiae* (Wong and Wolfe 2005), each discussed in detail below. Such arrangements may facilitate synchronized expression of pathway components and support efficient utilization of purine-derived metabolites as nitrogen and, in some cases, carbon sources (Osbourn and Field 2009).

A well-characterized example is the *puc* system of *Bacillus subtilis*. Most genes involved in purine degradation are located within a chromosomal cluster and are organized into distinct transcriptional units (Schultz et al. 2001). These genes encode proteins involved in urate transport, xanthine oxidation, urate conversion to allantoin, and downstream degradation of allantoin-derived intermediates. The *pucJ* and *pucK* genes encode components of the urate transport system, whereas *pucA–E* are required for functional xanthine dehydrogenase activity. The conversion of urate to allantoin involves the products of *pucL* and *pucM*, and *pucH* encodes allantoinase. The *pucF* and *pucG* gene products participate in subsequent reactions involving allantoate and ureidoglycine. Although *pucI*, which encodes an allantoin permease, is not physically located within the main chromosomal cluster, it belongs to the same regulatory network (Ma et al. 2016).

The expression of these genes is controlled by PucR and is sensitive to nitrogen availability. Under nitrogen-limiting conditions, pathway induction enables *B. subtilis* to use purine-derived compounds as alternative nitrogen sources. By contrast, expression is reduced when preferred nitrogen sources are abundant. Pathway intermediates, including urate, allantoin, and allantoate, also influence PucR-dependent regulation (Schultz et al. 2001). This organization illustrates how genomic clustering and transcriptional control can be integrated to balance purine degradation with the preservation of metabolites required for nucleotide biosynthesis. A distinct but functionally related organization is found in *Klebsiella pneumoniae*. In this organism, the *hpx* gene cluster (hpxP/hpxC/hpxA/hpxB/hpxK/hpxJ) encodes proteins involved in the uptake and sequential degradation of hypoxanthine to allantoin (De la Riva et al. 2008; Guzmán et al. 2011). The cluster includes transporters and enzymes that act at different stages of the pathway. The products of *hpxD* and *hpxE* participate in the conversion of hypoxanthine to urate, whereas *hpxO* encodes a flavin-dependent urate oxidase. Additional genes encode proteins involved in the conversion of urate-derived intermediates and allantoin metabolism. The clustering of these genes supports their coordinated regulation and enables the organism to assimilate hypoxanthine as a nitrogen source.

In particular, hpxA encodes an allantoin racemase that interconverts (R)- and (S)-allantoin. Because the downstream allantoinase (HpxB/PuuE) is stereospecific for the (S)-enantiomer, while spontaneous, non-enzymatic decomposition of HIU and OHCUd generates racemic allantoin, this racemization step is thought to be required for efficient and complete utilization of allantoin derived from uric acid oxidation ((Pope et al. 2009). Rather than being inherently cytotoxic, (R)-allantoin represents a metabolic dead end in the absence of this racemase, since allantoinase cannot act on it directly. This creates a kinetic bottleneck that limits nitrogen recovery from the pathway rather than a documented source of direct toxicity, in contrast to upstream intermediates such as HIU (see Purine catabolism and uric acid degradation: an overview section).

Functional clustering is not restricted to prokaryotes. In *Saccharomyces cerevisiae*, six genes involved in allantoin utilization form the DAL cluster, located within a subtelomeric region of chromosome IX (Wong and Wolfe 2005). The cluster includes *DAL1*, *DAL2*, and *DAL3*, which encode enzymes responsible for the sequential degradation of allantoin to glyoxylate and urea; *DAL4*, which encodes an allantoin permease; *DAL7*, which encodes a malate synthase involved in glyoxylate utilization; and DCG1, which encodes a probable allantoin racemase, assigned on the basis of sequence similarity to characterized racemases, analogous in proposed function to bacterial hpxA (Wong and Wolfe 2005). Two additional genes involved in the pathway, *DAL5* and *DUR1,2*, are not physically located within the cluster. *DAL5* encodes an allantoate permease, whereas *DUR1,2* encodes a multifunctional enzyme responsible for urea degradation.

Comparative genomic analyses indicate that the DAL cluster arose relatively recently during yeast evolution through the relocation of genes that were previously dispersed across the genome (Wong and Wolfe 2005). This reorganization was associated with a shift in nitrogen utilization. While several yeast species retain the ability to import and oxidize urate, *S. cerevisiae* and *Naumovozyma castellii* lack functional urate permease and urate oxidase genes and instead rely on the uptake and degradation of allantoin. By bypassing the oxygen-dependent urate oxidation step, this metabolic configuration may favor growth under oxygen-limited conditions (Wang et al. 2025).

The genomic arrangement of DAL genes is also functionally relevant. Experimental inversion of *DAL2* in *S. cerevisiae* reduced the expression of *DAL2* itself and altered the expression of neighboring genes, including *DAL1* and *DAL4*. These changes impaired growth when allantoin was supplied as the nitrogen source (Naseeb and Delneri 2012). Thus, the biological significance of the DAL cluster is not limited to the physical proximity of its genes; their relative order can influence pathway expression and cellular fitness under nitrogen-limiting conditions (Leulliot et al. 2004). Table 1 summarizes and aligns the functional correspondences among these three systems, highlighting both the conserved logic of pathway organization (transport, oxidation, and sequential degradation) and the taxon-specific differences in gene nomenclature and cluster composition.

**Table 1:** Representative genomic organization of genes involved in purine and ureide catabolism. Comparison of genes associated with the *B. subtilis puc* system, the *K. pneumoniae hpx* cluster, and the *S. cerevisiae* DAL cluster. Genes are grouped according to their principal functions in transport and the sequential degradation of purine-derived metabolites. In *B. subtilis*, *pucI* belongs to the PucR regulon but is located outside the main chromosomal cluster. In *S. cerevisiae*, *DAL5* and *DUR1,2* participate in allantoin utilization but are not physically located within the DAL cluster

Pathway step or function	*B. subtilis*	*K. pneumoniae*	*S. cerevisiae*
Urate transport	*pucJK*	*hpxP*	–
Allantoin transport	*pucI*^1^	*hpxC*	*DAL4*
Hypoxanthine to urate	*pucABCDE*	*hpxDE*	–
Urate to allantoin	*pucLM*	*hpxO* and associated genes	–
Allantoin to allantoate	*pucH*	*hpxB*	*DAL1*
Allantoin racemization	–	*hpxA*	*DCG1*
Allantoate degradation	*pucF*	*hpxK*	*DAL2*
Ureidoglycine-associated reactions	*pucG*	*hpxJ*	–
Ureidoglycolate to glyoxylate	–	–	*DAL3*
Glyoxylate utilization	–	–	*DAL7*

^1^pucI belongs to the PucR regulon but is located outside the principal puc cluster

The genomic organization of purine degradation genes is more diverse than the classical *puc*, *hpx*, and DAL systems suggest. In *Escherichia coli*, for example, urate metabolism cannot be described by a single aerobic uricolytic cluster analogous to those of *B. subtilis* or *K. pneumoniae*. Instead, studies have identified distinct systems associated with urate uptake and degradation under anaerobic or microaerobic conditions (Xi et al. 2000). A formate-dependent pathway allows *E. coli* to use urate as a nitrogen source in the absence of abundant oxygen (Iwadate and Kato 2019). More recent studies have also characterized a reductive uric acid degradation pathway encoded by a broader gene cluster and distributed among anaerobic bacteria, including members of the gut microbiota (Liu et al. 2023; Ali 2025). These findings expand the known diversity of uric acid catabolism and indicate that alternative genomic architectures may support urate utilization in distinct ecological contexts.

Together, these examples demonstrate that genomic organization is an important dimension of uric acid catabolism. Physical clustering, transcriptional regulation, gene relocation, and alternative pathway architectures contribute to the adaptation of microorganisms to changes in nitrogen availability, oxygen concentration, and ecological niche. Comparison of these systems also illustrates how metabolic pathways can be reorganized at the genomic level while preserving core biochemical functions, a principle that extends beyond microorganisms to the selective retention and decay of uricolytic genes in hominoids, examined in the following sections.

## Ecological, evolutionary, and biomedical contexts of uric acid metabolism

The biological significance of uric acid metabolism extends beyond the catalytic properties of individual enzymes. Across different organisms, uricolytic pathways are embedded in distinct ecological and physiological contexts, ranging from nitrogen assimilation in plant–microbe symbioses to the regulation of serum urate levels in humans. These contexts have shaped the retention, loss, and reorganization of pathway components during evolution. In addition, recent studies indicate that the evolutionary history of uric acid catabolism may have consequences for human health, including susceptibility to hyperuricemia, gout, and potentially other disorders associated with urate metabolism.

### Ureide metabolism in plant–microbe interactions

In several tropical legumes, including soybean (*Glycine max*), uric acid degradation plays an important role in nitrogen assimilation and long-distance nitrogen transport. Within root nodules, atmospheric N_2_ is reduced to ammonia by nitrogen-fixing bacteria associated with the plant. The fixed nitrogen is then incorporated into purines and converted through purine oxidation and uric acid degradation into ureides, particularly allantoin and allantoate. These nitrogen-rich metabolites are transported from the nodules to other plant tissues, where they contribute to growth and metabolism (Kahn and Tipton 1997; Baral, Da Silva, and Izaguirre-Mayoral, 2016).

The interaction is metabolically reciprocal. While the bacteria provide fixed nitrogen to the plant, the plant supplies carbon sources, including dicarboxylic acids, that sustain bacterial metabolism within the specialized environment of the root nodule. As a result, flux through the ureide pathway is especially important in ureide-transporting legumes, where efficient processing of purine-derived metabolites supports nitrogen redistribution throughout the plant.

The high metabolic demand associated with this pathway also explains why several uricolytic enzymes were first isolated and characterized from soybean nodules. These tissues contain relatively high levels of proteins involved in urate-derived metabolite processing, facilitating the purification and biochemical characterization of enzymes such as HIUase (Kahn and Tipton 1997; Sarma et al. 1999).

This physiological context is relevant for understanding the evolution of pathway organization. When metabolic flux depends on the rapid conversion of unstable intermediates, the spatial and regulatory integration of consecutive catalytic activities may provide a functional advantage. Natural HIUase-OHCU fusion proteins represent one possible solution to this biochemical constraint.

### Ecological context of HIUase and OHCUd fusion proteins

As discussed in the Urah–Urad gene fusions as metabolic and evolutionary innovations section, the sequential conversion of 5-hydroxyisourate (HIU) to (S)-allantoin can be carried out by HIU hydrolase (*Urah*) and OHCU decarboxylase (*Urad*) as independent enzymes or, in plants, diatoms, and some bacteria, as a single naturally fused polypeptide sometimes referred to as allantoin synthase (Pessoa et al. 2010; Oh et al. 2018). The structural architecture of these fusions exemplified by the allantoin synthase of the diatom *Phaeodactylum tricornutum* (PDB ID: 5Z5M; Oh et al. 2018) is consistent with colocalization of consecutive catalytic activities rather than direct substrate channeling, as detailed in the Urah–Urad gene fusions as metabolic and evolutionary innovations section.

**Fig. 4:**
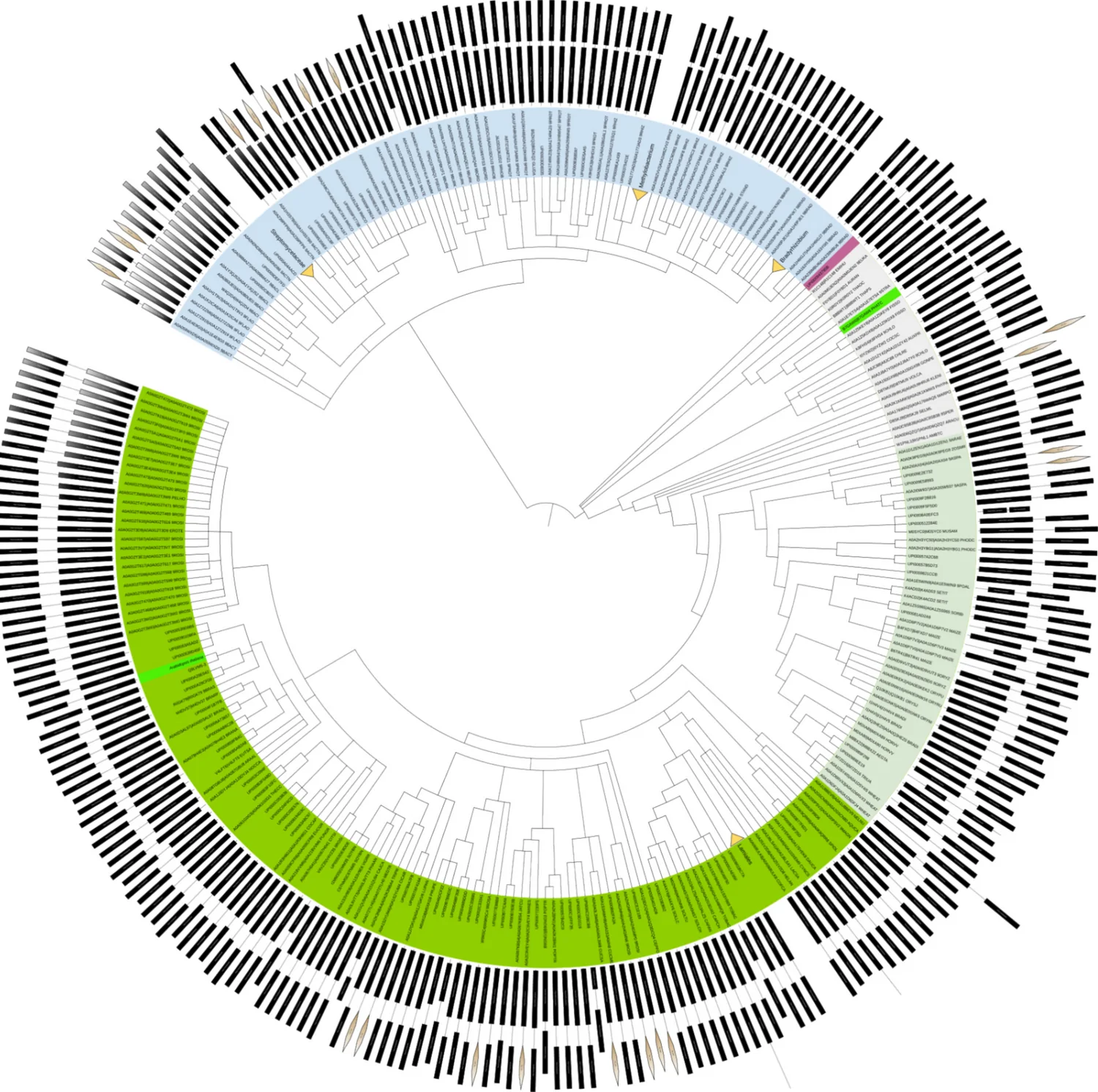
Phylogenetic distribution of HIUase-OHCUd fusion proteins. Schematic representation based on NCBI taxonomic topology (generated using iTOL), with domain architecture HIUase and OHCUd as separate proteins or as a single fused polypeptide, determined via SMART domain analysis of UniProt sequences mapped onto each branch. This tree reflects reference taxonomy rather than de novo sequence-based phylogenetic inference of urate oxidase; species were included based on the presence of at least one of these genes, as identified through UniProt. The occurrence of fused architectures in phylogenetically distant lineages is consistent with multiple independent fusion events. The occurrence of fused architectures in phylogenetically distant lineages is consistent with multiple independent fusion events. Taxonomic groups and protein architectures are indicated. The blue segments correspond to sequences from plant species that possess bifunctional enzymes in which HIUase and OHCU decarboxylase activities are fused into a single polypeptide chain, evidencing functionally conserved gene fusion events. The green segments, in turn, represent sequences of bacterial origin; those in a more intense (darker) shade correspond to nitrogen-fixing bacteria, while those in a lighter shade correspond to other bacteria

The ecological relevance of these proteins is particularly plausible in systems characterized by substantial flux through purine-derived metabolites. In plants, ureide metabolism contributes to nitrogen assimilation and transport. In soil-associated and nitrogen-fixing bacteria, urate degradation may support nitrogen recovery under nutrient-limited conditions. In some bacterial lineages, horizontal gene transfer has also been proposed as a mechanism contributing to the dissemination of fused architectures. However, the magnitude of the adaptive benefit provided by HIUase and OHCUd fusion under specific environmental conditions remains to be established experimentally. As shown in Fig. 4, fused architectures occur in both plant lineages and multiple bacterial clades, including nitrogen-fixing species. The dynamic compatibility between the fused domains, examined through elastic network modeling, is discussed in the Exploratory Analysis of Collective Dynamics in a Natural HIUase and OHCUd Fusion section.

### Exploratory analysis of collective dynamics in a natural HIUase and OHCUd fusion

The compatibility between fused enzyme domains is not determined exclusively by static structural features. Protein dynamics may also influence whether two domains can be combined without compromising folding, oligomerization, or catalytic function. Elastic network approaches provide a useful framework for exploring the collective motions permitted by a protein architecture, complementing the ecological and structural considerations outlined in the Ecological context of HIUase and OHCUd fusion proteins section.

To investigate the dynamic properties of a natural HIUase and OHCUd fusion, we performed elastic network analyses using the crystallographic structure of the *P. tricornutum* allantoin synthase (PDB ID: 5Z5M). Anisotropic and Gaussian network model calculations indicate that the HIUase and OHCUd domains undergo largely uncorrelated collective motions, consistent with a hinge-like organization in which each catalytic unit retains substantial conformational independence rather than being mechanically coupled to its partner domain. This dynamic decoupling reinforces the interpretation that the functional advantage of the fused architecture does not stem from direct substrate channeling or concerted conformational coupling between active sites, but rather from fixed stoichiometry and spatial proximity that favor rapid, coordinated turnover of the unstable HIU and OHCUd intermediates, a mechanism that would be particularly advantageous in the ecological contexts described in the Ecological context of HIUase and OHCUd fusion proteins section (high-flux ureide metabolism in plants and nitrogen-limited bacterial environments), where minimizing diffusional loss and non-enzymatic decomposition of reactive intermediates could confer a measurable, if not yet directly quantified, fitness benefit.

**Fig. 5:**
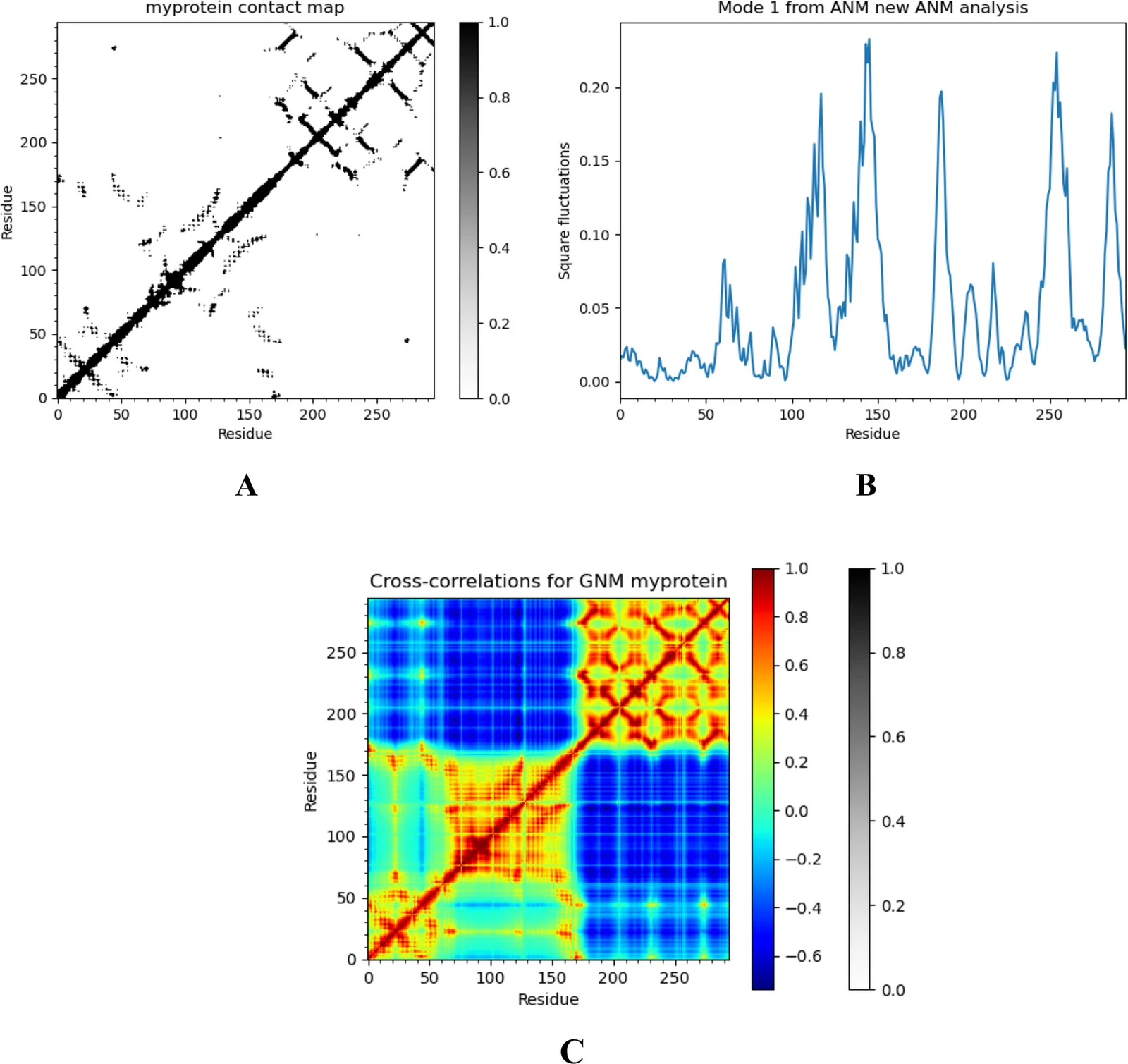
Collective dynamics analysis of protein 5Z5M using elastic network models. **A** Residue fluctuations associated with the lowest-frequency normal mode obtained from the anisotropic network model (ANM), highlighting regions of higher mobility and more rigid structural cores. **B** Residue–residue contact map derived from the crystallographic structure, illustrating the protein’s topological organization and long-range contacts that constrain collective motions. **C** Cross-correlation matrix of residue fluctuations calculated using the Gaussian network model (GNM), revealing dynamically correlated (positive correlation) and anticorrelated (negative correlation) regions, indicative of domain-level collective motions and long-range dynamic coupling. Colors represent the cross-correlation coefficient between the motions of residue pairs, ranging from +1.0 (dark red) to approximately −0.6 (dark blue). Dark red values, including the main diagonal, indicate strongly correlated motion (residues moving in the same direction); yellow-green values near zero indicate weak or no correlation; blue values indicate anticorrelated motion (residues moving in opposite directions). The comparatively weak correlation between these two blocks (off-diagonal regions) indicates largely independent collective motion between the two catalytic domains, consistent with the hinge-like organization described in the main text

The analysis revealed regions of differential mobility and long-range dynamic coupling within the fused architecture (Fig. 5). The residue fluctuation profile highlights comparatively rigid regions and more flexible segments, while the contact map illustrates the topological constraints imposed by the three-dimensional structure. The cross-correlation matrix identifies groups of residues with correlated or anticorrelated motions, providing an initial view of the collective dynamics of the fusion protein.

To determine whether catalytic residues show distinct dynamic behavior, we mapped the active-site residues of each domain (identified by sequence alignment) onto the GNM/ANM profiles. Catalytic residues in both domains displayed intermediate absolute mobility (35th–70th percentile of all residues), with the exception of the C-terminal catalytic Tyr296, whose elevated fluctuation likely reflects a chain-terminus artifact rather than genuine local flexibility. Notably, active-site residues showed higher mean cross-correlation with the rest of their own domain (OHCUd: *r* = 0.41; HIUase: *r* = 0.43) than the domain-wide average (*r* = 0.35 and 0.34, respectively), indicating that catalytic sites move as an integral, well-coupled part of their host domain rather than as dynamically isolated loops. Conversely, active-site residues showed stronger anticorrelation with the opposite domain (OHCUd active site vs. HIUase: *r* = −0.42; HIUase active site vs. OHCUd: *r* = −0.44) than the domain-wide interdomain average (*r* = −0.34), indicating that the mechanical independence between domains is most pronounced precisely at the catalytic sites. This pattern supports a model in which each active site is dynamically integrated with and moves coherently with its own catalytic domain, while remaining largely decoupled from the partner domain’s motion, consistent with independent catalytic turnover at spatially proximal but mechanically autonomous active sites.

Because this analysis is based on a single structure, it should be interpreted as exploratory rather than as evidence that equivalent dynamic patterns are universally conserved across HIUase-OHCUd homologs. Nevertheless, the results provide a starting point for comparative studies involving fused proteins, isolated domains, and non-fused homologs. Such comparisons could help determine whether recurrent gene fusion events are associated with the preservation of mechanically compatible motions across domains.

From a broader perspective, the analysis illustrates that evolutionary innovation may depend not only on the preservation of structural scaffolds and active-site geometry, but also on dynamic compatibility between protein modules. The HIUase-OHCUd system therefore provides a useful model for examining the relationship between gene fusion, oligomeric organization, and collective protein motions.

### Evolutionary consequences of uricase loss in hominoids

The evolutionary history of uric acid metabolism in hominoids differs significantly from that in organisms with a complete uricolytic pathway. In humans and other hominoids, mutations accumulated in the urate oxidase gene (UOX), resulting in the loss of a functional uricase enzyme. Since UOX catalyzes the first step in converting urate to allantoin, its inactivation disrupted the enzymatic degradation of uric acid and may have reduced selective pressure on downstream pathway components, including HIUase- and OHCU decarboxylase-related genes (Keebaugh and Thomas 2010; Ramazzina et al. 2006).

The molecular mechanisms behind UOX pseudogenization have been studied extensively. Marchetti et al. (2016) tested the hypothesis that uricase inactivation occurred gradually through accumulating mutations that progressively impaired enzyme function. They analyzed the effect of the F222S substitution and found that this variant had a much higher Michaelis constant than the reference enzyme, with *K*_m_ values of about 280 μM and 11 μM, respectively. These results support a progressive decline in uricase efficiency before complete loss of function.

This conclusion is consistent with ancestral sequence reconstruction studies showing that mammalian uricase activity declined gradually and continuously over approximately 80 million years, from around 96 Ma to complete pseudogenization near the onset of the Miocene (~15–20 Ma), coinciding with global climatic cooling (Kratzer et al. 2014). Notably, loss of uricase activity is not restricted to the hominoid lineage: it has occurred independently in multiple, phylogenetically unrelated groups, including birds, terrestrial reptiles, and cyclostomes (Oda et al. 2002), paralleling the pattern of convergent, independent gene fusion events described for Urah–Urad elsewhere in this review (Urah–Urad gene fusions as metabolic and evolutionary innovations section). Together, these observations reframe uricase pseudogenization not as an isolated hominoid-specific event, but as part of a broader, recurrent evolutionary pattern of relaxed selective constraint on uricolytic pathway components across vertebrates.

Several adaptive hypotheses have been proposed to explain why increased serum urate concentrations may have been tolerated or favored during hominoid evolution. These include the antioxidant properties of uric acid and its possible role in maintaining blood pressure under dietary conditions with limited sodium availability (Keebaugh and Thomas 2010). Other hypotheses have been discussed, but the relative importance of these mechanisms remains uncertain.

The absence of functional uricase leads to the relatively high serum urate concentrations seen in humans. While this trait may have provided advantages in certain ancestral environments, it also increases susceptibility to hyperuricemia and gout. Evolutionary changes in urate transporter 1 (URAT1) appear to have further promoted urate retention in primates. Tan et al. (2016) found that primate URAT1 orthologs have greater affinity for urate than rodent orthologs, supporting the idea that renal urate reabsorption coevolved with the loss of uricase activity.

The physiological consequences of this evolutionary path remain relevant in modern populations. Nonfunctional URAT1 variants are linked to lower serum urate levels and may reduce the risk of hyperuricemia and gout. In a genetic epidemiological study, a substantial proportion of hypouricemia cases with serum urate concentrations ≤2.0 mg/dL were attributed to nonfunctional URAT1 variants (Suo et al. 2025). These findings highlight the delicate balance between retaining urate as a potentially beneficial metabolite and the pathological effects of excessive accumulation.

### The gut microbiome as a compensatory uricolytic system

Although humans lack a complete functional uricolytic pathway, the gut microbiome may partially compensate for the absence of host uricase activity. Liu et al. (2023) demonstrated that anaerobic gut bacteria can metabolize uric acid through pathways distinct from the classical oxygen-dependent uricolytic route.

Microbial degradation of urate can produce xanthine or lactate, as well as short-chain fatty acids such as acetate and butyrate. These processes contribute to urate elimination and may influence serum urate concentrations. The findings support the concept that host and microbial metabolisms function as an integrated system, in which bacterial activities can partially replace enzymatic functions lost during host evolution.

This compensatory role of the gut microbiota also broadens the ecological interpretation of uric acid metabolism. The evolutionary loss of a host pathway does not necessarily eliminate the corresponding biochemical capacity from the organismal system as a whole. Instead, metabolic functions may be redistributed between the host and its associated microorganisms.

### Residual uricolytic genes and emerging biomedical questions in humans

Although humans lack a complete functional pathway for the enzymatic conversion of urate to downstream metabolites, genomic loci related to uric acid degradation can still be identified. These include UOX, URAHP, URAD, and ALLC, corresponding to urate oxidase-, HIUase-, OHCU decarboxylase-, and allantoicase-related regions, respectively (Cunningham et al. 2022).

The biological status of these loci should be considered carefully. The presence of a genomic region does not necessarily imply expression of a stable protein or retention of enzymatic activity. Nevertheless, potentially protein-coding transcripts have been reported for URAD and ALLC. Transcriptomic analyses indicate comparatively higher URAD expression in gastrointestinal tissues, whereas ALLC transcripts have been detected in tissues including tibial nerve and testis (Rodrigues et al. 2024). These observations raise questions about whether remnants of the ancestral uricolytic pathway may retain physiological functions unrelated to, or only partially overlapping with, their original metabolic roles.

A potentially relevant observation was reported by Giaccherini et al. (2023), who identified a significant association between the intronic variant 13q12.2-rs9581957-T and increased risk of pancreatic ductal adenocarcinoma. This variant is in linkage disequilibrium with rs9579139, a potentially deleterious missense variant of URAD. Although the functional consequences of these variants remain unclear, the association suggests that URAD-related molecular processes may have biomedical relevance.

Precise structural localization of the rs9579139-linked missense substitution would require mapping onto the human URAD structure (Rodrigues et al. 2026), which is beyond the scope of the current analysis. Nevertheless, we favor the hypothesis that a surface-exposed location is plausible and would be consistent with a role for URADd in protein–protein interactions independent of its residual catalytic function potentially connecting to the broader possibility, raised elsewhere in this review, that uricolytic pathway components may participate in higher-order molecular assemblies. We acknowledge that this remains speculative: to our knowledge, no study has systematically investigated URADd protein–protein interactions, interactome data, or surface-interface mapping in human tissues. This scarcity of direct evidence is itself notable and underscores an important gap in the field. We propose that interactome studies (e.g., affinity purification-mass spectrometry, yeast two-hybrid, or proximity-labeling approaches) applied to human URADd represent a valuable and currently unexplored avenue for testing this hypothesis, particularly given its potential relevance to the reported association with pancreatic ductal adenocarcinoma risk.

Recent studies on urate oxidation provide additional context for investigating this question. Even at concentrations commonly considered physiological, uric acid can undergo oxidation mediated by plasma peroxidases, generating a urate radical. Subsequent reactions may produce urate hydroperoxide and HIU. These reactive intermediates can affect cellular redox balance and promote covalent modification of lysine residues in proteins, a process termed uratylation (Dempsey et al. 2025).

The potential relationship between residual URAD activity, urate-derived reactive intermediates, and protein uratylation remains unknown. At present, there is no direct evidence that URAD modulates uratylation or contributes mechanistically to pancreatic tumorigenesis. However, the reported association between variants linked to URAD and pancreatic ductal adenocarcinoma provides a rationale for future biochemical and cellular studies.

An important open question is whether URAD encodes a stable and catalytically active human protein under specific physiological or pathological conditions. If so, it will be relevant to determine its subcellular localization, substrate specificity, oligomeric state, structural stability, and possible involvement in pathways triggered by urate oxidation. These questions illustrate how the evolutionary remnants of uric acid metabolism may offer new perspectives on human physiology and disease.

Recent experimental work demonstrated that recombinant human OHCUd decarboxylase adopts a folded α-helical structure and retains residual catalytic activity, although substantially reduced relative to zebrafish OHCUd decarboxylase (Rodrigues et al. 2026).

Together, these examples demonstrate that uric acid metabolism must be understood across multiple biological scales. In plants and microorganisms, the pathway contributes to nitrogen assimilation and ecological adaptation. In hominoids, the loss of uricase reshaped urate homeostasis and created new physiological constraints. In humans, the gut microbiome partially compensates for the absence of host uricase, while residual uricolytic genes raise questions about the persistence of ancestral functions and the emergence of new biological roles.

### Structural conservation of URAD across mammals

Following the pseudogenization of UOX in hominoids, selective pressures acting on downstream uricolytic enzymes were expected to decrease. Under this scenario, progressive sequence divergence and structural degeneration could be anticipated for URAD orthologs. To investigate whether such changes occurred, comparative sequence and structural analyses were performed across representative mammalian URAD proteins.

**Fig. 6:**
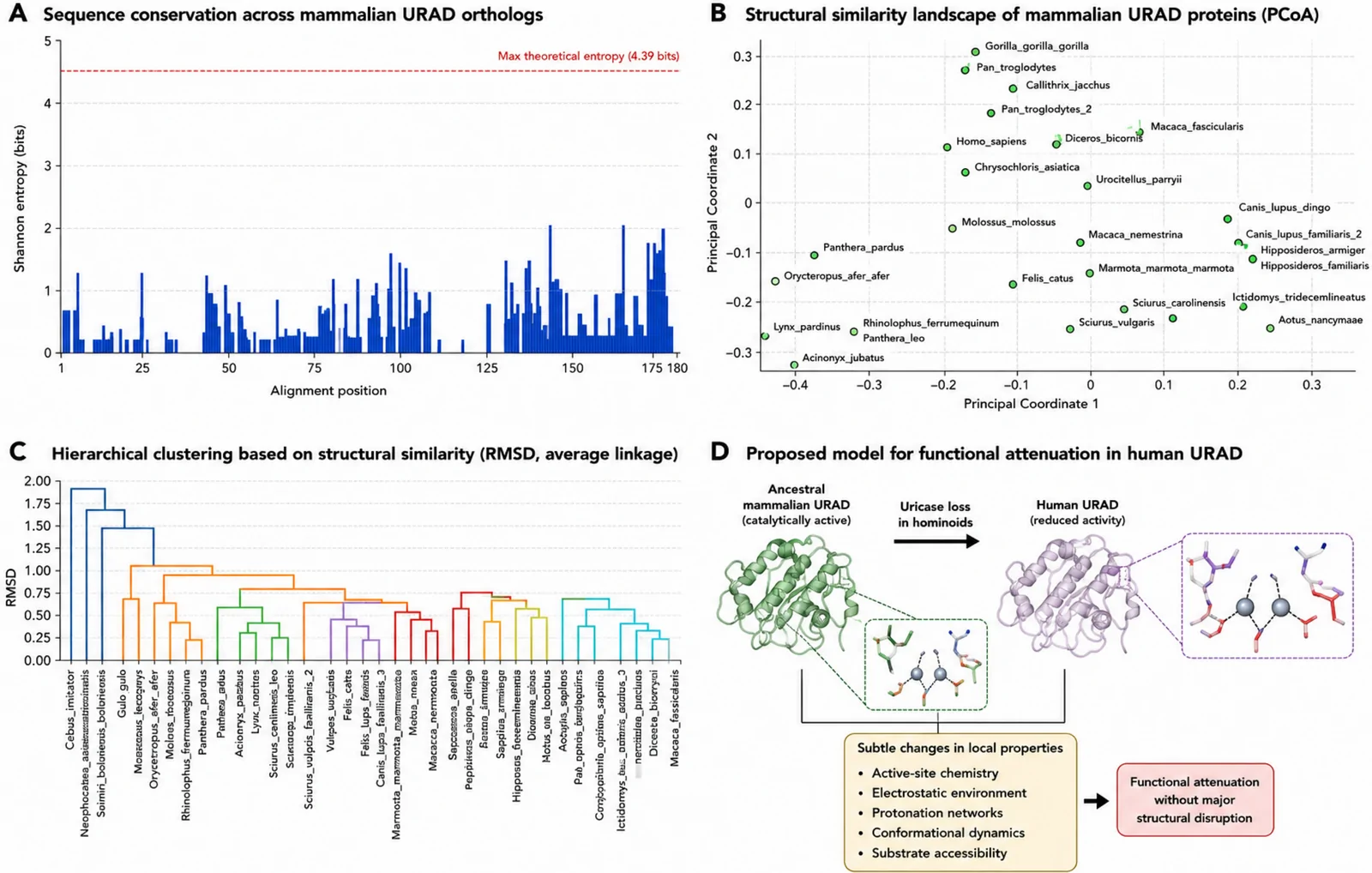
Evolutionary conservation and structural divergence of mammalian URAD proteins. **A** Shannon entropy analysis calculated from the multiple sequence alignment reveals strong sequence conservation across mammalian URAD orthologs. **B** Principal coordinate analysis derived from pairwise RMSD values shows that structural similarity largely follows phylogenetic relationships, with human URAD clustering with other hominids. **C** Hierarchical clustering based on RMSD confirms the preservation of the ancestral fold across mammals. **D** Proposed evolutionary model suggesting that the reduced catalytic performance of human URAD results from subtle changes in local active-site chemistry, electrostatics, proton-transfer networks, and conformational dynamics rather than from global structural degeneration

Shannon entropy analysis revealed low variability throughout most alignment positions, indicating that mammalian URAD proteins remain highly conserved despite their distinct evolutionary histories (Fig. 6A). Regions displaying the lowest entropy values correspond predominantly to the structural core and residues associated with catalytic function, suggesting the persistence of strong evolutionary constraints on the overall architecture.

Structural comparisons based on pairwise RMSD values further demonstrated that URAD proteins occupy a highly restricted structural space. Principal coordinate analysis showed that orthologs cluster according to phylogenetic relationships, with *Homo sapiens* grouping closely with other hominids, including *Pan troglodytes* and *Gorilla gorilla* (Fig. 6B, C).

Notably, the human protein does not emerge as a structural outlier. Instead, it remains embedded within the same structural landscape occupied by catalytically active orthologs. These observations indicate that functional attenuation in human URAD likely occurred without major disruption of the ancestral fold (Fig. 6D).

**Fig. 7:**
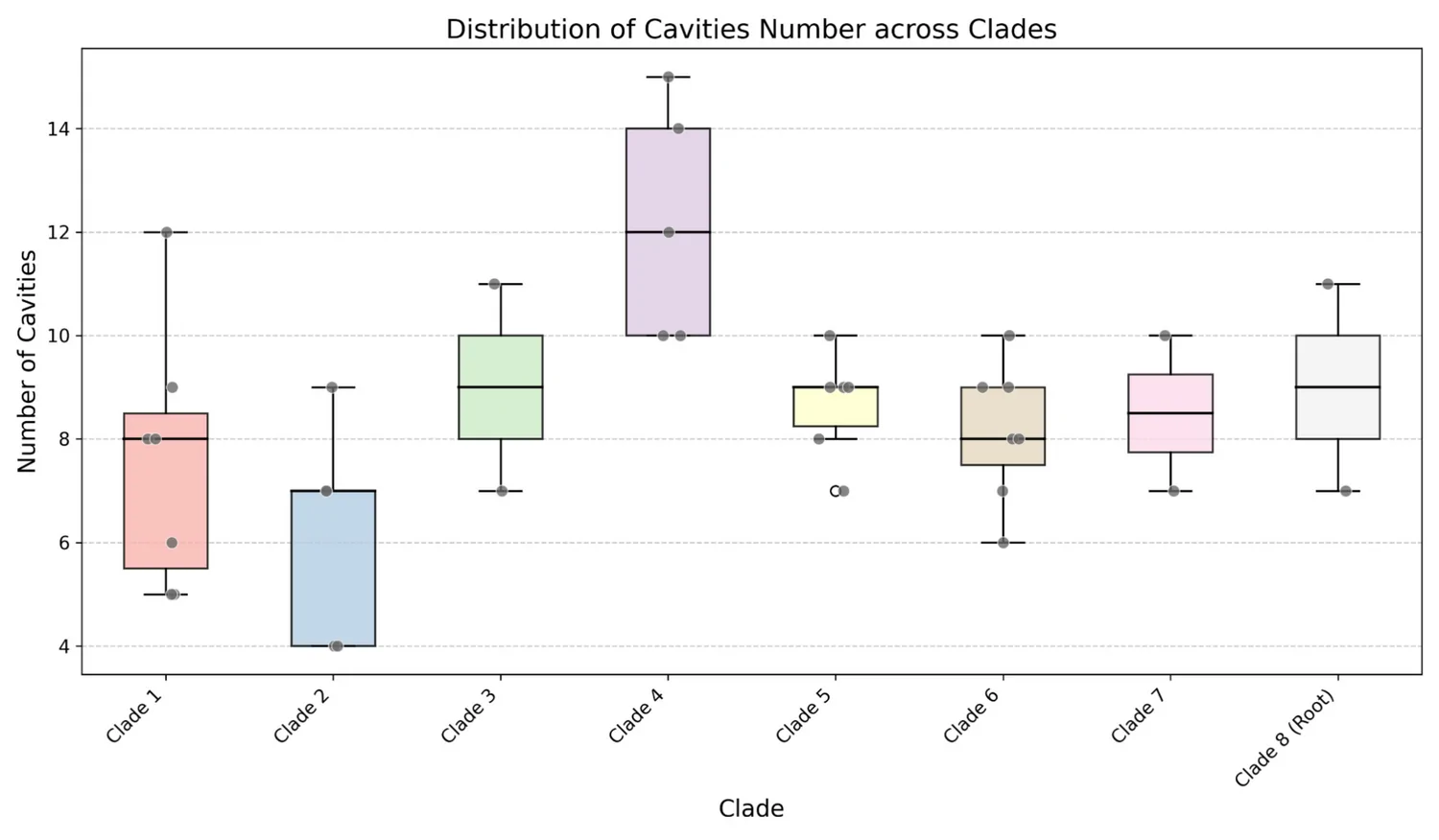
Distribution of predicted active-site cavity number across OHCUd decarboxylase clades. Box plots showing the number of cavities identified in structural models of OHCUd orthologs, grouped by clade (clades 1–8; clade 8 corresponds to the root/ancestral grouping). Boxes indicate the interquartile range, horizontal lines represent the median, and whiskers extend to the most extreme values within 1.5× the interquartile range; individual data points are overlaid as gray circles, and open circles denote outliers. Clades correspond to well-supported taxonomic groupings in most cases—clade 1 (Catarrhini), clade 2 (Platyrrhini), clade 3 (Chiroptera), clade 4 (Felidae), clade 5 (Caniformia), and clade 8 (Afrotheria, the outgroup/root)—but two clades (6 and 7) include taxonomically distant species (*Diceros bicornis* grouping with Sciuridae in clade 6; a cetacean and a bat co-occurring in clade 7), indicating that structural similarity in Urad does not strictly track phylogenetic relatedness. Clade 4 (Felidae) shows the broadest distribution and highest median cavity number (~12), while clade 2 (Platyrrhini) shows the widest low-end range (4–9); the remaining clades cluster within a comparatively narrow intermediate range (~8–9). The largest-volume cavity in each model was assumed to correspond to the catalytic pocket, consistent with its size and position relative to conserved active-site residues, though this was not confirmed residue-by-residue for every ortholog. Cavities were detected using pyKVFinder with default parameters (minimum volume cutoff ≈ 5 Å^3^); across all 36 models, individual cavity volumes ranged from 5.2 to 803.1 Å^3^ (median 19.2 Å^3^), and the majority (57%) were below 20 Å^3^, indicating that most detected cavities correspond to small surface clefts rather than large, functionally relevant pockets. Accordingly, cavity count should not be interpreted as a direct proxy for function, and this analysis does not assess whether adjacent cavities merge into continuous access tunnels; such an assessment would require dedicated tunnel-detection approaches (e.g., CAVER or MOLE). No formal statistical test (e.g., Kruskal–Wallis) was applied to compare cavity number across clades; the analysis is presented as descriptive and exploratory, analogous to the elastic network analysis in the Exploratory analysis of collective dynamics in a natural HIUase and OHCUd fusion section

Consistent with this structural conservation, active-site cavity analysis (see Fig. 7 in the From evolutionary biophysics to protein design: integrating structural and genomic evidence section) placed human URAD within the same range observed for other hominid orthologs in clade 1 (5–12 cavities), rather than as a numerical outlier, further supporting the absence of gross structural degeneration despite reduced catalytic activity.

Together, these findings support a model in which loss of enzymatic performance results primarily from subtle alterations in active-site chemistry, electrostatic environment, protonation networks, conformational dynamics, or substrate accessibility rather than from global structural degeneration.

## From evolutionary biophysics to protein design: integrating structural and genomic evidence

Over recent years, our work has systematically combined comparative genomics, structural biology, and biophysical analysis to investigate the evolutionary fate of enzymes involved in uric acid catabolism and to extract general principles relevant to protein design. As an initial step, we revisited the human uricolytic pathway using genome-wide and transcript-level analyses, demonstrating that genes encoding uricase, HIUase, OHCUd decarboxylase, and allantoicase are not simply absent from the human genome but persist as pseudogenes, long non-coding RNAs, or protein-coding transcripts with tissue-specific expression patterns (Rodrigues et al. 2024). This finding challenged the prevailing view of a completely lost pathway and revealed that molecular remnants of uric acid metabolism remain embedded in human genomic and regulatory networks.

Building on this genomic foundation, we have examined the structural and biophysical properties of human OHCUd, a distinct enzyme from urate oxidase, acting two catalytic steps downstream in the same pathway (Fig. 1B). As discussed in the Evolutionary consequences of uricase loss in hominoids section, pseudogenization of *UOX* is proposed to have relaxed selective constraint on the downstream enzymes HIUase and URADd, since the substrates of these enzymes are no longer generated once urate oxidation ceases; this reflects relaxed purifying selection on genes whose products became functionally dispensable, rather than any direct physical or mechanistic dependency between the OHCUd and uricase proteins themselves. Consistent with this prediction, URADd retains a stable α-helical fold closely resembling catalytically active OHCU decarboxylase orthologs from other species, despite exhibiting substantially reduced catalytic activity relative to its zebrafish counterpart (Rodrigues et al. 2026). This pattern with retention of native fold despite functional decline is consistent with the well-established principle that loss of enzymatic activity does not necessarily entail loss of structural integrity (Todd, Orengo, and Thornton, 2002); robust protein scaffolds can persist long after selective pressure on catalytic function is relaxed, preserving architectures compatible with folding, oligomerization, and molecular interactions. Beyond descriptive and comparative analyses, our group has actively applied these evolutionary and biophysical principles to guide rational protein engineering. A central example is our evolution-based protein engineering study (Lemos et al., 2024) in which we demonstrated functional interconversion between transthyretins (TTRs) and 5-hydroxyisourate hydrolases (HIUases). Phylogenetic reconstruction and sequence conservation analyses identified residues that are locally conserved within each subfamily but divergent between them. Rather than targeting global sequence similarity, the design strategy focused on residue subsets shaping cavity geometry, volume, and electrostatic properties, a key biophysical determinants of ligand specificity. Systematic substitution of these evolutionarily informed positions yielded in silico variants predicted to partially switch functional characteristics while preserving overall structure and electrostatic potential consistent with their redesigned functions. Importantly, functional switching was achieved without large-scale structural rearrangements, instead exploiting the intrinsic robustness and modularity of the ancestral scaffold. This result reinforces a central theme of this review: evolutionary conservation of protein architecture creates permissive landscapes for functional innovation, whether driven by natural selection or rational design.

Consistent with the hypothesis that functional attenuation may involve subtle changes in active-site architecture rather than global structural degeneration, cavity analysis across OHCUd orthologs revealed uneven distribution of predicted cavity number among clades, with clade 4 showing the broadest range and highest median values (Fig. 7). This heterogeneity suggests that active-site cavity architecture is not uniformly conserved across the OHCUd phylogeny, potentially reflecting clade-specific adaptations in substrate accessibility or catalytic microenvironment.

It should be noted that clade sample sizes were uneven, ranging from two orthologs (clades 3, 7, and 8) to seven (clades 1 and 6); consequently, the apparent distributions for the smaller clades should be interpreted as preliminary rather than statistically robust. Clades largely correspond to well-supported taxonomic groupings: clade 1 (Catarrhini), clade 2 (Platyrrhini), clade 3 (Chiroptera), clade 4 (Felidae), clade 5 (Caniformia), and clade 8 (Afrotheria, the outgroup/root), although clades 6 and 7 each include taxonomically distant species (*Diceros bicornis* clustering with Sciuridae in clade 6; a cetacean and a bat co-occurring in clade 7), indicating that structural similarity in URADd does not strictly track phylogenetic relatedness. Notably, clade 4 corresponds entirely to Felidae (*Panthera pardus*, *Panthera leo*, *Felis catus*, *Acinonyx jubatus*, and *Lynx pardinus*), indicating that the elevated cavity number in this group reflects a lineage-specific structural feature rather than a diffuse phylogenetic trend.

In this context, natural *Urah–Urad* fusion genes are particularly relevant for both biomedicine and protein engineering. Humans lack a functional uricolytic pathway, and current enzyme replacement therapies for hyperuricemia rely on administering multiple enzymes with distinct stability and immunogenicity profiles. Natural bifunctional enzymes such as HIUase-OHCUd provide a blueprint for designing single-chain, multi-enzymatic biotherapeutics that could simplify formulation, improve pharmacokinetics, and minimize the accumulation of toxic intermediates. The recurrent evolutionary convergence toward Urah–Urad fusion exemplifies how biophysical constraints, metabolic efficiency, and evolutionary innovation intersect, offering both mechanistic insight and inspiration for rational protein design.

Taken together, this body of work shows how evolutionary biophysics can be applied to practical design strategies. By tracing how nature assembles, dismantles, and repurposes uric acid catabolic enzymes across taxa, we identify molecular architectures that are both evolutionarily validated and biophysically feasible. These principles form a foundation for developing multi-enzymatic constructs and therapeutic strategies to restore or modulate uric acid metabolism, demonstrating how integrative analyses of genomics, structure, and function can guide the design of biologically grounded protein systems.

## Conclusion

The evolutionary history of uric acid degradation illustrates how metabolic pathways are shaped by the interplay between gene duplication, loss, and functional divergence. From its ancestral configuration, successive duplications and gene fusions generated enzymes with new activities and regulatory properties, resulting in the remarkable taxonomic diversity observed today. The conservation of structural scaffolds, as seen in HIUase and transthyretin (Carrijo de Oliveira et al. 2021; Prapunpoj et al. 2000; Yamauchi, Kasai, 2018; Zanotti et al. 2006), reveals how existing molecular frameworks can be co-opted for novel functions. The convergent fusion of Urah and Urad into single-chain allantoin synthases in plants, diatoms, and some bacteria (Pessoa et al. 2010; Oh et al. 2018) illustrates a complementary strategy, in which domain colocalization addresses the instability of consecutive reaction intermediates. Divergence in families such as UGLYAH, AAH, and UAH further exemplifies adaptive specialization to distinct ecological contexts (Puggioni et al. 2014; Shin et al. 2014). At the genomic level, the emergence of gene clusters in bacteria and yeast reflects optimization under nutrient-limited conditions (Guzmán et al. 2011; Ma et al. 2016; Wong and Wolfe 2005; Xi et al. 2000), whereas in plants, integration into the ureide pathway underpins symbiotic nitrogen assimilation (Baral, Da Silva, and Izaguirre-Mayoral, 2016). In hominoids, pseudogenization of *UOX* relaxed purifying selection on downstream enzymes, leading to elevated serum uric acid levels (Keebaugh and Thomas 2010; Ramazzina et al. 2006), an adaptation that once may have conferred physiological advantages (Keebaugh and Thomas 2010; Tan et al. 2016) but now contributes to disease susceptibility (Dempsey et al. 2025; Suo et al. 2025). Notably, recent biophysical characterization indicates that human OHCUd retains its ancestral fold despite substantially reduced catalytic activity (Rodrigues et al. 2026). This dissociation between structure and function suggests that the enzyme may be trapped in a low-entropy conformational basin, even if structurally conserved, dynamically restricted, and catalytically silent, a hallmark of pseudoenzymes and evolutionary vestiges that retain their scaffold for possible non-catalytic roles even as selective constraint on catalysis relaxes. Interestingly, recent evidence also suggests that symbiotic gut microbes can metabolize uric acid, thereby contributing to the maintenance of lower serum uric acid levels and reducing the risk of hyperuricemia (Liu et al. 2023).

These evolutionary trajectories underscore how metabolic systems remain dynamic, retaining traces of their past while acquiring new roles. Beyond its evolutionary significance, the uric acid pathway continues to influence physiology through interactions with the microbiome, redox signaling, and potentially tumorigenesis, as suggested by the association between URAD-linked variants and pancreatic ductal adenocarcinoma risk (Giaccherini et al. 2023). The same evolutionary principles that shaped this pathway also inform its translational potential, offering a rational basis for engineering single-chain, multi-enzymatic biotherapeutics for hyperuricemia and related disorders. Understanding these processes not only deepens our insight into enzyme innovation and regulatory evolution but also highlights new avenues for investigating the interface between metabolism, conformational dynamics, protein design, and human health.

## Data Availability

No datasets were generated or analyzed during the current study.

## Footnotes

1 Uricotelic organisms—including birds, reptiles, and insects—excrete excess nitrogen predominantly as uric acid rather than ammonia (ammonotelic) or urea (ureotelic), an adaptation that minimizes water loss due to uric acid’s low solubility.
